# Long term survival and outcomes in patients with paraneoplastic neurologic syndromes

**DOI:** 10.3389/fimmu.2024.1466704

**Published:** 2024-11-18

**Authors:** Sapir Bar Mucha, Ayal Rozenberg, Lilach Gutter Kapon, Alon Gorenshtein, Esther Ganelin-Cohen, Rachel Ben Hayun, Nataliya Yarovinsky, Shahar Shelly

**Affiliations:** ^1^ Department of Neurology, Rambam HealthCare Campus, Haifa, Israel; ^2^ Neuroimmunology Laboratory, Ruth and Bruce Rapaport Faculty of Medicine, Technion – Israel Institute of Technology, Haifa, Israel; ^3^ Neuroimmunology Laboratory, Rambam HealthCare Campus, Haifa, Israel; ^4^ Azrieli Faculty of Medicine, Bar-Ilan University, Safed, Israel; ^5^ Neuroimmunology Clinic, Institute of Pediatric Neurology, Schneider Children’s Medical Center of Israel, Petah Tikva, Israel; ^6^ Faculty of Medical and Health Sciences, Tel Aviv University, Tel Aviv, Israel

**Keywords:** paraneoplastic syndrome, cancer related neurological symptoms, encephalitis, diagnosis, long term follow-up, prognosis

## Abstract

**Objective:**

It is unknown whether delay in diagnosis affects morbidity reportedly in paraneoplastic syndromes (PNS). We aimed to explore various aspects of PNS, including prevalence, clinical characteristics, diagnostic criteria, and treatment outcomes.

**Methods:**

We studied n-PNS diagnosis between 2016 to 2023, and included only patients with positive onconeural antibodies, who developed cancer, and exhibited a recognizable PNS phenotype.

**Results:**

We identified 12 patients with positive Abs and co-occurring cancer, most prevalent PNS antibodies included anti-GAD65, anti-Recoverin and anti-Yo. The most common phenotypes were limbic encephalitis (n=5, 42%) and encephalomyelitis (n=4,33%). Cancer preceded neurological presentation in 6 cases. Among the 6 patients who initially presented with n-PNS, median time from neurological presentation to oncologic diagnosis was 73 days, as five of them (83%) were diagnosed with cancer during oncological evaluation prompted by the PNS diagnosis or suspicion. Lymphoma was the most frequent cancer (n=3, 25%), followed by lung cancer (n=2, 17%), and ovarian cancer (n=2, 17%). Among patients who received immunotherapy as n-PNS treatment (n=9, 75%), steroids were a part of the management at 78% (n=7). Another immunotherapy used included plasmapheresis (n=5, 55%) and steroid sparing immunosuppressant (n=2, 29%). Four (33%) patients had short term therapeutic benefit with improvement or stabilization at mRS ≤ 4. Median Disability-adjusted life years (DALYs), as disease burden value, was 13 years. Death occurred in 9 of the 12 patients, with most cases deaths attributed to cancer progression. Compering to the expected median survival by type and stage of tumor, from 9 deceased patients, 56% (n=5) died younger than expected. Median survival was 410 days (range 29-2738 days), and 152 days since the appearance of n-PNS (range 8-1434 days). There were no differences in survival between patients who initially presented with n-PNS versus cancer (p=0.39).

**Conclusion:**

In up to 8 years of follow up, there was no difference in mortality among patients who presented initially n-PNS. There was a significant decline in the quality of life, most face substantial disability and functional impairment long term.

## Graphical Abstract

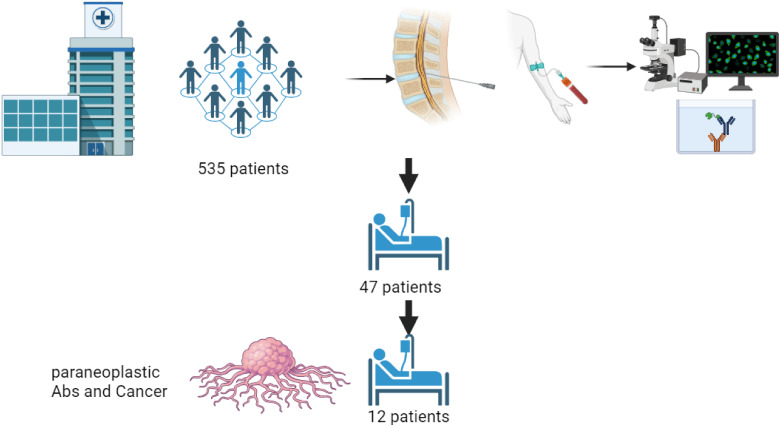

## Introduction

Paraneoplastic syndromes are infrequent clinical manifestations characterized as diverse autoimmune disorders, displaying a distinct set of clinical features that emerge alongside neoplastic disease process (1–5). Prior to the identification of novel antibodies, paraneoplastic syndromes were thought to affect between 1% and 7.4% of cancer patients (6). Recent advancements in antibody detection have revised these figures, showing that these syndromes now affect an estimated 10-15% of individuals with cancer (1, 7). Paraneoplastic syndromes can manifest as the initial clinical presentation of undetected cancer, during treatment for a newly diagnosed cancer, or as an indication of cancer relapse (1). These syndromes are associated with a range of affected systems, including neurologic, endocrine, dermatologic, musculoskeletal, hematologic, renal, and other systems (6).

The characteristics of neurological symptoms classified the syndrome as paraneoplastic neurologic syndrome (PNS). The most frequently associated with PNS tumors are lung carcinoma, breast cancer, ovarian cancer, and lymphomas (8). Awareness of clinical manifestation and diagnosis of PNS has greatly increased in recent years. In 2021, new diagnostic criteria for PNS were established by the PNS-Care panel (8). Epidemiology trends demonstrated that the incidence of PNS is increasing over time. This trend is likely attributable to increased awareness and the advancement of detection techniques, including the identification of numerous new neuronal antibodies and correlation to existing immunoglobulins like oligoclonal bands (9, 10).

Recent studies highlight a significant increase in morbidity and mortality among patients with PNS (11–13). These findings indicate that these patients often experience survival rates that fall short of expectations based on the type and stage of their cancer, affecting approximately two-thirds of cases (12). Furthermore, PNS patients are observed to accumulate higher total disability-adjusted life years (DALYs) compared to those afflicted with other diseases (11). Despite the diverse outcomes associated with PNS, research on their prevalence and specific risk factors, such as types of tumors, associated antibodies, phenotypes, and treatments, remains scarce with only a few studies addressing these issues (11–13).

In this study, our objective was to identify the risk factors associated with early mortality compared to the median survival expected based on the correlation with tumor type and stage (8). Additionally, we aimed to investigate the risk factors contributing to mortality specifically due to complications arising from PNS. To reach this goal, we made a comparison for many demographic and medical history parameters, clinical presentation parameters, test results, treatment management, quality of follow-up, and accepted scores such as modified Rankin Scale (mRS) and Clinical Dementia Rating (CDR).

## Methods

This is a retrospective database study conducted in a tertiary referral center Rambam HealthCare Campus (RHC). The RHC institutional review board approved this study, and all patients consented to the use of their medical records for research purposes.

### Cohort selection

**Figure 1:**
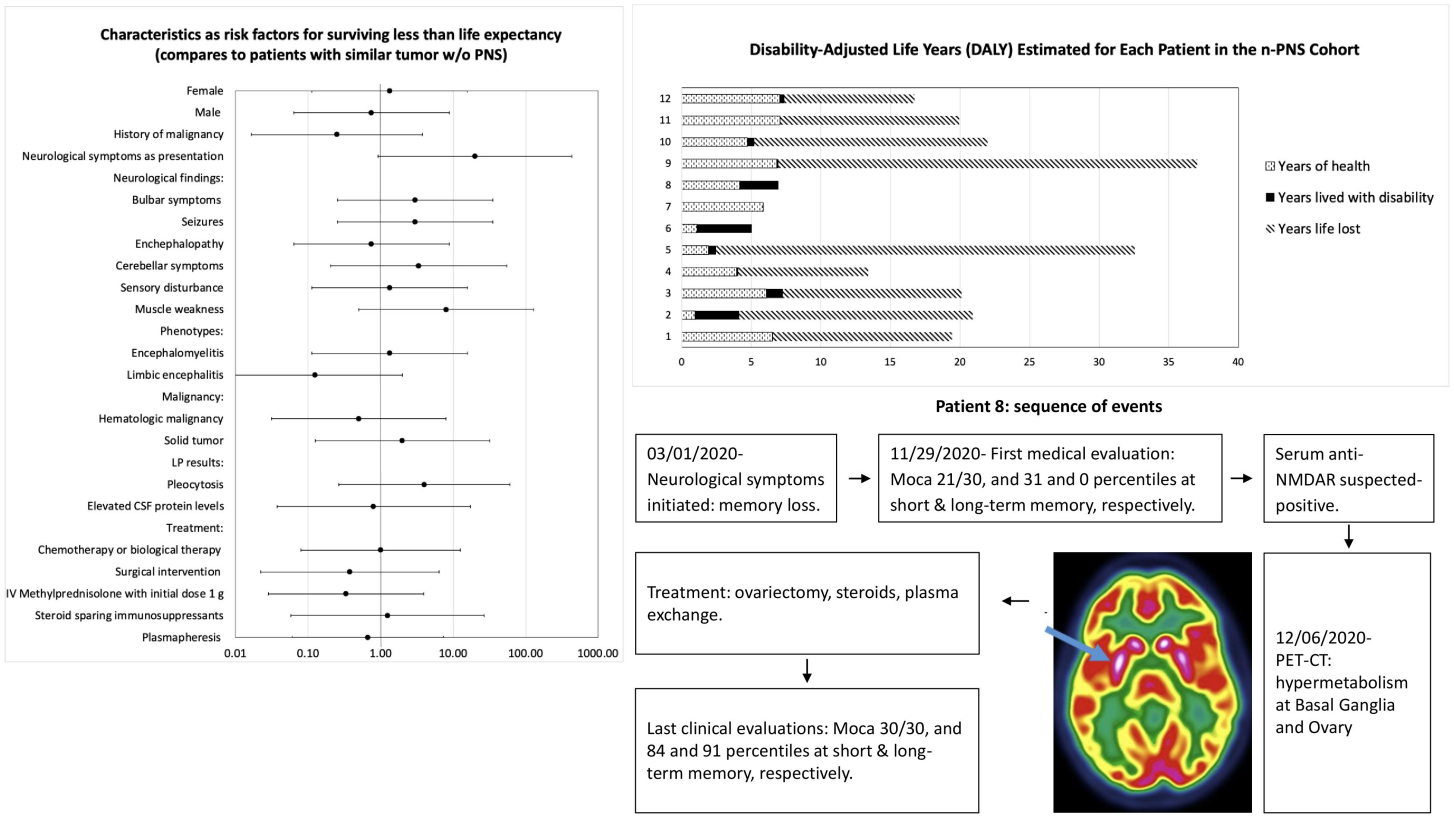
patients (n=535) underwent serum or CSF onconeural antibody panel testing at Rambam Health center between 1/11/2016-6/3/2023. We excluded 488 due to negative antibody test results. 47 patients had non-negative antibody test results included “suspected-positive” and “low-positive” results. 35 excluded due to lack of cancer diagnosis or recognizable PNS phenotype. 12 patients eventually developed cancer and exhibited a recognizable PNS phenotype.

We retrospectively identified all patients who underwent serum or CSF onconeural antibody panel testing at RHC 1/11/2016 to 6/3/2023. The presence of 18 unique antibodies were identified by immunofluorescence (IF) or immunoblot (IB) assays, as detailed in **Table 1**. **Figure 1** (flowchart) shows the study design. We included in the study patients who: diagnosed with either a confirmed malignancy or a probable malignancy with high certainty which could not be confirmed due to the patient’s fragile state; and exhibited a recognizable PNS phenotype as determined by a board-certified neurologist (**Table 2**). We excluded all patients with other causes of their symptoms.

**Table 1:** Tested antibodies.

Antibodies	Tested by IF or IB
Anti Ma2	**IB**
Anti Amphyphysin	**IB**
Anti CV2 (CRMP5)	**IB**
Anti GAD65	**IB**
Anti Hu (ANNA-1)	**IB**
Anti Recoverin	**IB**
Anti Ri	**IB**
Anti Sox1	**IB**
Anti Titin	**IB**
Anti Tr/DNER	**IB**
Anti Yo (PCA-1)	**IB**
Anti Zic4	**IB**
Anti AMPAR1	**IF**
Anti AMPAR2	**IF**
Anti CASPR2	**IF**
Anti GABA	**IF**
Anti LGI1	**IF**
Anti NMDAR	**IF**

The presence of 18 unique antibodies were extracted from serum or CSF and were identified by immunofluorescence (IF) or immunoblot (IB) assays as detailed. AMPAR, α-amino-3-hydroxy-5-methyl-4-isoxazolepropionic acid receptor; ANNA, antineuronal nuclear antibody; CASPR2, contactin-2 associated protein; CRMP5, collapsin response-mediator protein 5; DNER, Delta/Notch-like epidermal growth factor-related; GABA, Gamma-aminobutyric acid; GAD, glutamic acid decarboxylase; LGI1, leucine-rich glioma inactivated 1; NMDAR, N- methyl-D-aspartate receptor; PCA, Purkinje cell antibody.

**Table 2:** Test result summary for all included patients.

Patient number	The associated $antibody^{1}$	Positive at serum or CSF $panel^{1}$	Result	Phenotype	Cancer type	Coexistence antibodies	PNS-care score
PT-6 PT-10	anti-Yo (PCA-1) (n=2)	Serum (n=2)	positive (n=2)	RPCS (n=2)	Breast cancer (n=1) Ovarian carcinoma (n=1)	–	Definite (n=2)
PT-5	anti-Hu (ANNA-1) (n=1)	Serum (n=1)	positive (n=1)	EM (n=1)	SCLC (n=1)	anti GAD65 (serum, low-positive)	Definite (n=1)
PT-1	anti-NMDAR (n=2)	CSF (n=1)	suspected-positive (n=2)	LE (n=2)	TCC (n=1)	anti-Yo (CSF, positive) anti-ZIC4 (CSF, positive)	Definite (n=2)
PT-8		Serum (n=1)			Ovarian teratoma (n=1)	anti-Recoverin (serum, suspected-positive)	
PT-7	anti-Sox1 (n=2)	CSF (n=1)	suspected-positive (n=1)	LE (n=2)	Prostate cancer (n=1)	–	Probable (n=2)
PT-4		Serum (n=1)	low-positive (n=1)		squamous cell lung carcinoma (n=1)	anti-GAD65 (serum, low-positive)	
PT-3	anti-Ma2 (n=1)	Serum (n=1)	high-positive (n=1)	Sensory neuropathy (n=1)	B cell lymphoma (n=1)	–	Probable (n=1)
PT-9	anti-CV2 (CRMP5) (n=1)	CSF (n=1)	positive (n=1)	EM (n=1)	carcinoma with unknown origin (n=1)	–	Probable (n=1)
PT-11	anti-GAD65 (n=1)	CSF (n=1)	positive (n=1)	LE (n=1)	Leukemia (n=1)	–	Possible (n=1)
PT-12 PT-2	anti-Recoverin (n=2)	CSF (n=1) Serum (n=1)	suspected-positive (n=2)	EM (n=2)	T cell lymphoma (n=2)	–	Possible (n=2)

Data for each PNS associated antibody is detailed: incidence out of 12 patients, from which specimen it was extracted, strength of the result, PNS phenotype, cancer type, coexistence antibodies at a patient, and PNS-care scores that were calculated. ANNA, antineuronal nuclear antibody; CRMP5, collapsin response-mediator protein 5; CSF, cerebrospinal fluid; GAD, glutamic acid decarboxylase; NMDAR, N- methyl-D-aspartate receptor; PCA, Purkinje cell antibody; SCLC, small-cell lung cancer.

Clinical information obtained through an electronic record retrieval system until 31/12/2023. Patients labeled as smokers if smoking is mentioned in their medical records in the past or at present. The time of PNS diagnosis is defined as the time of receiving the oncological antibodies test result. The time of cancer diagnosis was defined as the time of biopsy for patients who underwent biopsy with pathological findings. In one case, the patient did not undergo a biopsy procedure due to oncologic guidance that recommended initiating treatment without biopsy, in that case, the time of cancer diagnosis was defined as the time imaging was performed.

### Immunoblot and immunofluorescence assays

Autoantibodies were detected in patient serum or cerebrospinal fluid by transfected cell-based immunofluorescence assay (EUROIMMUN, Lubeck, Germany) (14).

### Outcome evaluation

The modified Rankin Scale (mRS) was used to assess clinical outcome. The mRS is an ordinal 6-point scoring system that measures neurological disability and has been widely applied to evaluate acute and long-term outcomes in patients with autoimmune encephalitis. mRS score was calculated at 3 time points: before onset, at onset, and at last follow-up. Oncological and neurological outcomes, as reported in the last medical records, were also mentioned in this study and included cancer status and neurological symptoms status as assessed by the attending physicians. Mortality and causes of mortality were reported as noted in medical records. Survival in each case was also compared to the expected median survival by tumor type and stage. We have tested the following variables, morbidity: We examined factors contributing to severe morbidity, as defined by a modified Rankin Scale (mRS) score exceeding a specified threshold (mRS > 3). Therapeutic Benefit: Our analysis will assess determinants of therapeutic benefit, characterized by either improvement or stabilization of patients with a pre-treatment mRS score of ≤4 or improvement in cases with a pre-treatment mRS score of 5 (15). Tumor-Related Survival: We investigated factors influencing tumor-related survival, including the correlation between early mortality and the expected median survival based on tumor type and stage. Mortality due to PNS complications: factors contributing to mortality resulting from complications related to paraneoplastic syndromes (PNS).

Another modality used for morbidity and mortality estimations was a hybrid model of disability-adjusted life years (DALYs), calculated as the summation of incidence-based years of life lost (YLL) and prevalence-based years lived with disability (YLDs) (16). YLL was defined as the standard expected years of life lost based on the age at death from the World Health Organization Global Burden of Disease. Using extensive population-based survey data, Global Burden of Disease (GBD) has provided nonfatal burden estimates including disability weights for neurologic conditions (17, 18). Our data referred to the categories motor impairment, motor and cognitive impairment, epilepsy, major depressive disorder, and intellectual disability, which were applied to individuals in our PNS cohort. For each patient in the cohort, YLD was defined as the cumulative years from neurologic syndrome onset to death, last follow-up, or end of the study period, multiplied by the assigned disability weight.

### Statistical analysis

Group differences were established using Wilcoxon statistics between the means of continuous variables. Survival was analyzed, using Kaplan–Meier, and Cox regression analysis following further grouping of our patients into those who died or survived. P< 0.05 was regarded as statistically significant.

## Results

### Patients’ selection

Out of 535 patients, 47 (9%) tested “positive”, “suspected-positive” or “low-positive” for PNS-related antibodies, but only 12 who developed cancer with a clear PNS phenotype were included for analysis. According to the PNS-care score by Graus et al. (5), 42% (n=5) received a definite diagnosis, 33% (n=4) probable, and 25% (n=3) possible. Two possible cases exhibiting lymphoma and presenting with antibodies not listed in Graus et al. criteria were included in the analysis due to their clinically evident neurological phenotypes, which are associated with PNS. All specific test results are shown in **Table 2**. The follow-up period from symptoms onset until the last medical record or death ranged from 29 days to 7.5 years.

### Demographics and clinical data

Demographic data and medical history were collected and summarized in **Table 3**. 42% of all patients were females. The median age at symptoms presentation was 70 years (range 29-81), and the median age at the last follow-up or at death was 73.5 years (range 32-81). Among all patients, almost half (42%, n=5) were smokers at presentation. The history of other malignancy diseases was noted at 33% (n=4) which were bladder transitional cell carcinoma (TCC), renal cell carcinoma, chronic myelogenous leukemia, and larynx malignancy. Coexisting Immunosuppression was reported at 17% (n=2) while in one case due to azathioprine as inflammatory bowel disease treatment, and in the other case due to treatment for a kidney transplant.

**Table 3:** Demographic, clinical, testing and outcomes characteristics of PNS patients.

Category/outcomes:	Survived less than life expectancy (n=5*)	Survived as expected (n=6)	w/o Therapeutic Benefit (n=8)	with Therapeutic Benefit (n=4)	Total (n=12)
Gender, Male	3	4	5	2	7
Median age at presentation, years	74	70	75	61.5	70
Medical history:					
Cardiovascular risks	5	5	8	3	11
Coexisting Immunosuppression	0	1	0	1	1
History of malignancy	1	3	4	0	4
Common neurological examination findings:					
Muscle weakness	4	2	5	1	6
Sensory disturbance (include pain)	2	2	3	1	4
Cerebellar symptoms (include ataxia and vertigo)	2	1	3	1	4
Enchephalopathy (include cognitive and memory decline, delirium and psychiatric manifestations)	3	4	5	3	8
Seizures	3	2	5	0	5
Bulbar symptoms (include dysarthria, dysphagia and diplopia)	3	2	4	2	6
Syncope	1	0	1	0	1
Phenotypes:					
Limbic encephalitis	1	4	3	2	5
Encephalomyelitis	2	2	3	1	4
Rapidly progressive cerebellar syndrome	1	0	1	1	2
Sensory neuropathy	1	0	1	0	1
Malignancy:					
Solid tumor	4	4	5	3	8
Hematologic malignancy	1	2	3	1	4
Neurological symptoms as first presentation	4	1	4	2	6
**LP results (n):**	4	6	7	4	11
Median CSF protein	51	108	75	94	70
Median CSF leukocyte cells	34	6	17	5	9
Treatment (n/n total):					
Chemotherapy or biological therapy	3/5	3/5	5/8	2/3	7/11
Surgical intervention	1/5	2/5	2/8	1/3	3/11
Radiotherapy	0/5	1/5	1/8	0/3	1/11
IV Methylprednisolone with initial dose 1000 mg	2/5	4/6	3/8	3/4	6/12
Initial dose 500 mg	1/5	0/6	1/8	0/4	1/12
Steroid sparing immunosuppressants	1/5	1/6	1/8	1/4	2/12
Plasmapheresis	2/5	3/6	3/8	2/4	5/12
Survival:					
Median DALYs	17	11.3	13	1.4	13
Median survival, as time from first presentation to last medical record or death, months	5	33.5	9	40	13
Median survival from neurological symptoms to last medical record or death, months	5.9	2.5	2.8	36	5.1

Demographic and clinical data, followed by testing, treatment and survival data, comparing patients who survived less than life expectancy vs. those who lived as expected by tumor type and stage. Additionally, the same parameters were compared between patients who were represented without therapeutic benefit vs. those who achieved therapeutic benefit.

* One patient follow-up was shorter than life expectancy while she was still alive, and hence her data was not included.

Psychiatric symptoms were aggression, anxiety and depression.

The most common neurological complaints were generalized weakness and diplopia, reported in 25% of patients each. Other neurological complaints at presentation included muscle weakness, dysphagia, walking difficulty, paresthesia, loss of consciousness, delirium, muscle pain, vertigo, headache, generalized seizures, cognitive decline, aggression, fall and head injury, and memory decline. Each of these symptoms was reported by at least one patient, with frequencies ranging from 8% to 17%. Neurological examination findings are summarized in **Table 3** with the most common findings including muscle weakness in 50% (n=6) of patients, followed by seizures and delirium in 42% (n=5) of patients. All patients in this study were classified as having “high-risk” phenotypes according to the updated criteria for PNS (5). The most observed phenotype was limbic encephalitis, present in 42% of patients (n=5), followed by encephalomyelitis, observed in 33% of patients (n=4). The less commonly observed phenotypes were rapidly progressive cerebellar syndrome, present in 17% of patients (n=2), and sensory neuropathy, observed in 8% of patients (n=1).

PNS was the initial presentation in half of the patients, while the other half had oncologic symptoms prior to the onset of PNS symptoms. The median time from neurological presentation to oncologic diagnosis was 73 days (range 23-373). Among the six patients who initially presented with PNS, five (83%) were diagnosed with cancer during oncological evaluation prompted by the PNS diagnosis or suspicion. In one case, (P6) neurological symptoms were initially misdiagnosed as a psychiatric disorder, leading to a delayed diagnosis of cancer, a year after the onset of neurological symptoms (positive antibodies for anti-Yo). Among the 6 patients whose first presentation was oncological symptoms, data about the time of oncological symptoms initiation was available in 4 patients. Median time from oncological symptoms initiation or cancer diagnosis to neurological symptoms initiation was 664 days (range 36-2728), and to PNS diagnosis was 973.5 days (range 69-2737).

### Cancer diagnoses

**Table 2** categorizes cancer diagnoses by PNS antibody type. Hematological cancer was the most frequent with the following positive antibodies: anti-Recoverin (n=2), anti-GAD65 (n=1), and anti-MA2 (n=1). Data about ancillary tests that led to cancer diagnosis was available for 11 patients, and from 10 of them, a biopsy was done. The most common findings observed at radiology imaging were lymphadenopathy at 91% of cases, skeletal lesions at 45%, and lung consolidation or lesion at 36%. Positive biopsies with malignant histopathological findings were identified in 9 patients. At one patient (P11), the biopsy was not indicative of malignancy, probably due to clinical misdiagnosis. Noteworthy, in cases in which PNS was the first presentation, the median time from neurological presentation to first body screening imaging was 48 days (range 4-372), and the median time to biopsy was 59 days (range 23-289). In terms of oncologic staging, most patients (75%, n=9) were found to have advanced disease.

### Ancillary testing for PNS diagnosis

CSF analysis showed elevated protein levels in 9 out of 11 cases (median CSF protein 70 mg/dl, range 32-232 mg/dl). Patients with normal levels of CSF protein levels had antibodies for NMDAR and Sox1. Pleocytosis was reported at 64% (n=7) of patients (median of 9 cells/mm3, range 0-208 cells/mm3). Patient (P9) with extreme pleocytosis of 208 cells/mm3 had antibodies for CV2. Pathological CSF cytology was reported at 36% (n=4) and showed monoclonal lymphocytosis in one case, polyclonal lymphocytosis in two cases, and pleocytosis with increased red blood cells in one case (P9). The median time from neurological presentation to first brain imaging was 0 days (range 0-262 days), and only two had significant delays (P2 at 91 days and P8 at 262 days due to delay in patient consumption of medical services).

Additional tests were performed including EEG in 6 cases and EMG at 4 cases. Four cases showed exceptional EEG recordings showed slow background activity and in one case epileptiform discharges were obtained. Moreover, these four cases also showed abnormal EMG recording findings which were one or more of the following: axonal sensory and motor polyneuropathy (n=2), demyelinating neuropathy (n=2), ganglionopathy (n=1), and myopathy (n=1). The median time from neurological presentation to the first EEG was 49 days (range 1-334), and to the first EMG was 4 days (range 2-314).

### Therapy and medical follow-up

Data on cancer treatment was available for 11 patients. Of these, 27% (n=3) did not receive treatment due to poor prognosis (n=2) or undiagnosed progression and sudden clinical deterioration (n=1). The remaining 8 patients received treatment with a median initiation time of 29 days (range 0-68 days). Chemotherapy or biological therapy was the most common treatment (n=7), followed by surgery (n=3), and radiotherapy (n=1). Stable or responsive disease was observed in 38% (n=3) of treated patients, while 63% (n=5) showed disease progression. Among the 3 patients who underwent surgery, only one showed improvement in PNS symptoms post-operatively. In terms of chemotherapy and neurological symptoms, 3 patients received chemotherapy prior to neurological symptoms, with only one patient developing symptoms shortly after, fifteen days after treatment initiation.

Data on PNS treatment was available for 12 patients. Of these, 25% (n=3) did not receive PNS treatment due to proximity to death (n=2) or misdiagnosis (n=1). Among the 9 patients treated for PNS, 67% (n=6) initiated treatment before receiving positive antibody results, with a median time of -10 days (range -23 to 14 days). Steroids were used in 78% (n=7) of cases, with protocols including 1000mg (n=6), and 500mg (n=1) initial doses of IV Methylprednisolone, as a first-line therapy it was given in 5 cases; and Prednisone at a dose of 60 mg as a first-line therapy (n=1). Steroid sparing immunosuppressants or biological treatment, as additional treatment lines, were given in 2 cases, including Rituximab, Cyclophosphamide, and azathioprine. Plasmapheresis was administered in 5 cases, with 2 as a complementary treatment to steroids.

The follow-up period from symptoms onset until last medical record or death ranged from 29 days to 7.5 years. Out of 11 patients with available oncological follow-up data, proper oncological follow-up visits were formed at 45% (n=5); lack of follow-up due to recent oncologic diagnosis with poor prognosis was reported at 18% (n=2); and lack of proper follow-up was reported at 36% (n=4). The reason for all non-optimal follow-up visits was poor compliance for scheduling or attending routine follow-up visits (n=4), and even poor compliance for further ambulatory evaluation when abnormal findings emerged (n=3).

Of the 12 patients included in the study who had available neurological follow-up data, only 25% (n=3) received proper neurological follow-up visits. Lack of follow-up was reported in 42% (n=5) of patients due to a recent PNS diagnosis with poor prognosis, while 33% (n=4) of patients reported lack of proper follow-up. The reasons for non-optimal follow-up visits included poor compliance for scheduling or attending routine follow-up visits (n=1), poor compliance for further ambulatory evaluation when abnormal findings emerged (n=1), misdiagnosis (n=2), and/or lack of communication between medical units (n=1).

### Outcomes and survival analysis

**Figure 2:**
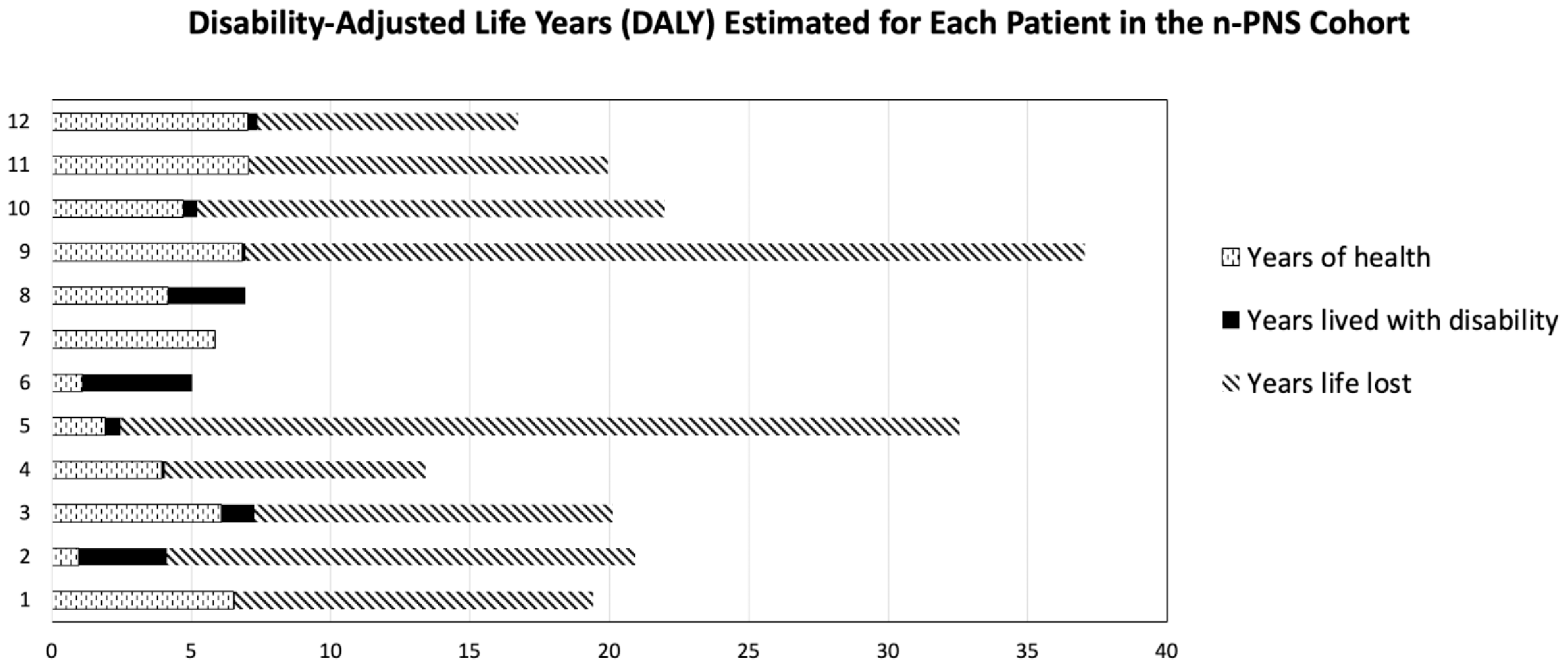
Disability-adjusted life years (DALYs) for each of the 12 patients, based on total years of life lost (YLL) plus years lived with disability (YLD).

**Figure 3:**
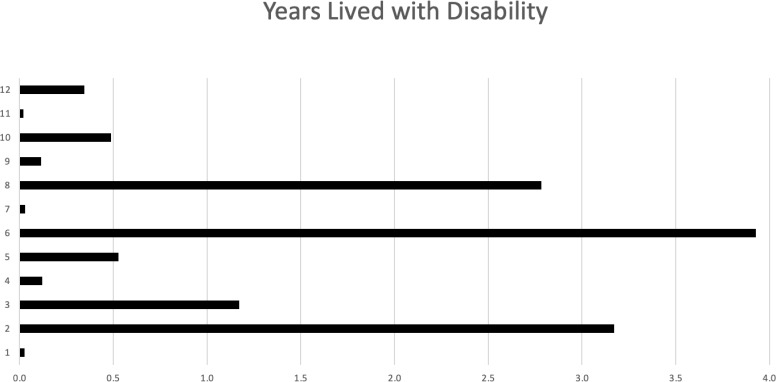
Years lived with disability (YLD) for each of the 12 patients.

Disease burden was calculated by the disability-adjusted life years (DALYs) model (**Figure 2**). Total DALYs for 12 patients with PNS and cancer were 156.4 years, based on total years of life lost (YLL) for patients dying between June 2018 and May 2023 (n=9) of 151 years, plus years lived with disability (YLD) for all patients (n=12) of 5.4 years. Median DALYs were 13 (range 0.008–30), median YLL were 12.8 (range 9.3–30.1), and median YLD were 0.18 (range 0.008–2.58), with individual DALYs, **Figure 3**. Final oncological status was available for 10 patients. 30% (n=3) of them showed a decrease in cancer size, with complete remission reported in 2 cases, while 70% (n=7) showed an increase in size and dissemination of cancer. Neurological symptoms progression as reported in medical records were available for all patients. 42% (n=5) of them showed stability or improvement in neurological symptoms from the worst presentation to the last medical record, while 58% (n=7) showed worsening symptoms. Stable or improved symptoms were reported in 56% (n=5), one patient (P8) showed full recovery with normal neurological examination and 30/30 at the Montreal Cognitive Assessment score, 2 patients showed partial recovery, and 2 patients had stable symptoms. Out of these patients, 2 patients were treated with one modality (plasmapheresis or Methylprednisolone alone), while others were treated with several modalities.

Out of all patients, 33% (n=4) had a therapeutic benefit, two patients had improvement at mRS (P2) had an initial score of 4 and final score of 1; P8 had an initial score of 2 and final score of 0), and two patients had unchanged mRS (P6 had an initial and final score of 3; P7 had an initial and final score of 1). The PNS associated antibodies in patients who were presented with therapeutic benefits were anti-Recoverin, anti-Yo, anti-Sox1, and anti-NMDAR.

Death occurred in 75% (n=9) out of all patients, with 67% (n=6) of deaths attributed to cancer progression. The causes of death were one or a combination of the following: respiratory failure in (n=4), multisystem failure in (n=3), septic shock in (n=2), pneumonia (n=2), and unknown cause (n=3). Median survival, as time from first presentation to last medical record or death, was 410 days (range 29-2738 days). Median survival from neurological symptoms initiation to last medical record or death, was 152 days (range 8-1434 days).

To isolate the decline in survival due to PNS only, survival from the first presentation was compared to the expected median survival by type and stage of tumor. All living patients (n=3) had yet to reach the expected survival of their tumors. Of the remaining patients (n=9), 56% (n=5) died younger than the expected median survival, and the rest (n=4) exceeded it. Notably, the survival of three individuals exceeded the expected median by 2 times. Their associated antibodies were anti-Recoverin with T cell lymphoma, multiple antibodies (anti-Yo, anti-ZIC4, anti-NMDAR) with TCC, and anti-GAD65 with leukemia. **Table 4** details the survival by tumor diagnosis and the respective expected median survival. There were no statistical differences between patients who were presented with cancer versus PNS, p=0.36.

**Table 4:** Survival in PNS cases compared against expected median survival by tumor type and stage.

Tumor	Associated Antibody	Number of cases	Survival (months) *=living	Expected median overall survival (months)	Sources
Extensive stage SCLC	anti-Hu	1	6	8-13 10	Clinical and translational oncology (2020) 22:245-255 (24)
Squamous cell lung carcinoma stage 4	anti-Sox1	1	2	8	Lung cancer 133 (2019) 96-102 (26)
Gynecological malignancies:					
Breast cancer stage 3a HER2 positive	anti-Yo	1	47*	Over 84	Lancet oncology (vol 15) 2014; 640: 647 (27)
Ovarian serous adenocarcinoma stage 3	anti-Yo	1	5	44	Gynecologic oncology 147 (2017); 243:249 (28)
Ovarian teratoma dermoid cyst bilateral	anti-NMDAR	1	33*	Non applicable	Journal of Neuro-Oncology (2019) 141:431-439 (12)
Hematologic malignancies:					
T cell lymphoma stage 3	anti-Recoverin	2	31*, 59 (peripheral)	31 (peripheral)	Peripheral: Annals of oncology 25 (2014); 2211:2217 (29)
B cell lymphoma marginal zone low grade	anti-Ma2	1	14	96-120 (108)	Best Practice & Research Clinical Haematology 30 (2017) 84-91 (30)
Leukemia- AML	anti-GAD65	1	12	12	Blood, 18 April 2013, volume 121 number 16 (30)
Other:					
Prostate cancer local, high risk	anti-Sox1	1	0.3*	Non applicable	J Clin Oncol 39 (2021); 1234:1242 (31)
Transitional cell bladder cancer, papillary urothelial carcinoma. high grade	anti-NMDAR	1	90	50	Am J Surg Pathol, December 2004, volume 28 number 12 (31)
Carcinoma of unknown primary origin, poorly differentiated carcinoma	anti-CV2	1	1	18	Oncotarget, 2017, volume 8 number (32)

*living patient.

**Figure 4:**
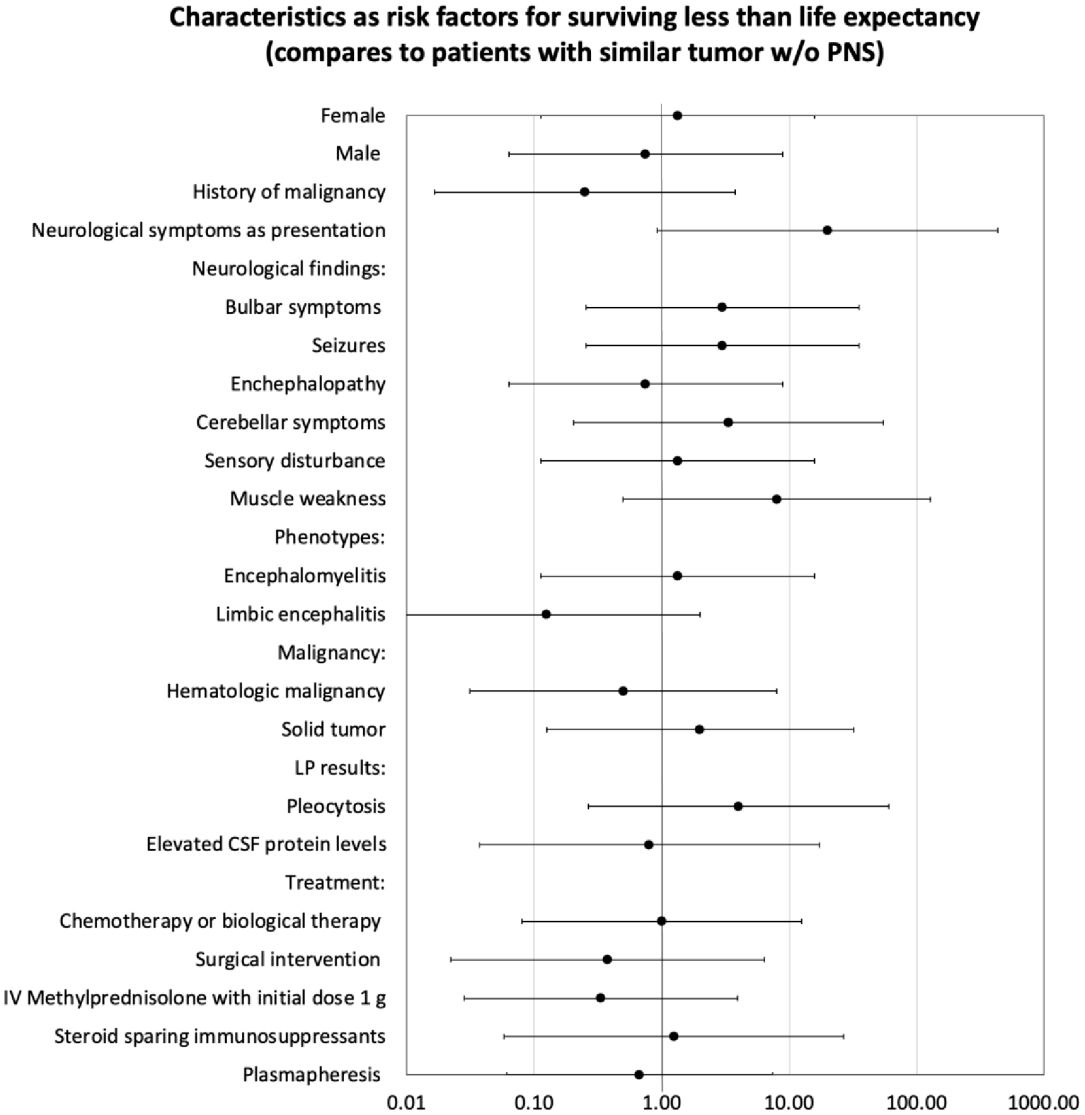
Analysis characteristics as risk factors for surviving less than life expectancy by odds ratio.

At last, analysis was performed on all demographic, clinical, testing and outcomes that was collected, in order to seek risk factors for surviving less than life expectancy at patients with PNS, as detailed at **Figure 4**. Statistically, we did not find any parameter that can be defined as risk factor for poor outcome.

## Discussion

PNS are rare with diverse clinical manifestations. We aimed to explore various aspects of PNS, including prevalence, clinical characteristics, diagnostic criteria, and treatment outcomes. We found the prevalence of PNS at 9% (47 out of 535), highlighting an upward trend likely due to improved recognition of many new neural autoantibody markers (19). The most common tumors in our study were lung carcinoma, breast cancer, ovarian cancer, and lymphomas, aligning with previous literature​​​​ (8). In our cohort, limbic encephalitis and encephalomyelitis were the most common phenotypes observed, underscoring the need to consider these conditions in the differential diagnosis of neurological symptoms in cancer patients.

In some of our cases, PNS was the initial clinical manifestation, leading to delayed cancer diagnoses as neurological symptoms were initially misinterpreted. This emphasizes the need for heightened clinical suspicion and awareness recognizing these symptoms. We showed n-PNS significantly impacted quality of life, with most patients (74%) having substantial disability and functional impairment, affecting their daily lives (**Figures 2**, **3**). Moreover, the mortality burden of PNS and cancer is considerable, with a survival rate below the median expected based on tumor type and stage for 42% of our cohort (**Table 4**). Outcomes varied depending on the antibodies present. We show the impact of specific antibodies on the prognosis of PNS patients. Anti-Recoverin, anti-NMDAR, and anti-GAD65 antibodies were associated with better survival and manageable neurological outcomes, suggesting a less aggressive disease course or more effective response to treatment. Conversely, anti-Yo and CV2 antibodies were linked to poorer outcomes, reflecting the severe nature of the diseases they accompany

Specifically, anti-Recoverin antibodies were associated with a notably better prognosis. Two patients with these antibodies had survival times exceeding the expected median by more than double, even with aggressive malignancies such as T-cell lymphoma. Cancer-associated retinopathy with recoverin-specific cytotoxic T lymphocytes can recognize and target cancer cells expressing recoverin​​ (20, 21). In our cohort, clinically patients with anti-Recoverin antibodies presented with encephalomyelitis instead of the more commonly associated cancer-associated retinopathy​​ (22). Anti-NMDAR antibodies are typically linked to anti-NMDAR encephalitis, often associated with ovarian teratoma (8).

In our study, two patients with these antibodies had normal CSF protein levels and experienced improved neurological outcomes with stable disease progression, suggesting these antibodies as indicators of manageable disease trajectories. Anti-GAD65 antibodies were observed in a patient who exceeded the median expected survival for leukemia, presenting with limbic encephalitis, a common manifestation associated with high anti-GAD65 titers (23). Patients with low titers of anti-GAD65, part of our inclusion criteria, had concurrent anti-Hu and anti-SOX1 antibodies, with notable findings of cognitive change. Anti-Yo antibodies were linked to poorer prognosis. Patients with these antibodies typically had aggressive cancers and significant neurological impairments. For example, two patients with anti-Yo antibodies showed shorter survival times compared to the expected median. The nature of anti-Yo, an intracellular neuronal antigen, leads to immune-mediated neuronal death, making therapeutic interventions less effective once significant neuronal loss occurs​​​​. Lastly, a patient with CV2 antibodies had extreme pleocytosis and experienced rapid cancer progression with worsening neurological symptoms, resulting in shorter survival. CV2 antibodies are strongly associated with small-cell lung cancer or thymoma (24), although our patient had poorly differentiated carcinoma involving multiple organs. We also compared protein CSF levels with patients who exceeded expected survival had higher median CSF protein levels compared to those with less favorable outcomes. Pleocytosis was more frequently observed when CSF studies were conducted early in the disease (25), suggesting these patients were in earlier stages and benefited from timely treatment interventions.

Several limitations should be acknowledged. The retrospective nature of our study might introduce selection bias, and the relatively small sample size limits the generalizability of our findings. Additionally, relying on medical records for data collection could lead to incomplete or missing information.

In conclusion, our study contributes to the evolving understanding of PNS by shedding light on its prevalence, clinical characteristics, and diagnostic challenges. PNS remains a complex and often underdiagnosed condition that demands increased awareness among healthcare providers. Multidisciplinary collaboration between neurologists, oncologists, and other specialists is crucial for improving the management and outcomes of PNS patients. Further research is warranted to delineate the risk factors contributing to morbidity and mortality in this patient population, ultimately guiding more effective treatment strategies. Future research should focus on conducting prospective studies with larger cohorts to further elucidate the risk factors associated with unfavorable PNS outcomes. Additionally, efforts should be made to establish standardized treatment guidelines that address the unique challenges posed by PNS.

## Data Availability

The original contributions presented in the study are included in the article/supplementary material. Further inquiries can be directed to the corresponding author.
